# Knowledge Level, Motivators and Barriers of Blood Donation among Students at Qatar University

**DOI:** 10.3390/healthcare9080926

**Published:** 2021-07-22

**Authors:** Amal Abdulsalam Ibrahim, Muammer Koç, Atiyeh M. Abdallah

**Affiliations:** 1 Department of Biomedical Sciences, College of Health Sciences, Qatar University, Doha 2713, Qatar; amal.ibrahim@qu.edu.qa; 2 Division of Sustainable Development, College of Science and Engineering, Hamad Bin Khalifa University, Qatar Foundation, Education City, Doha 5825, Qatar; mkoc@hbku.edu.qa; 3 Biomedical and Pharmaceutical Research Unit, QU-Health, Qatar University, Doha 2713, Qatar

**Keywords:** blood donation, young adults, motivators, barriers, awareness, students, Qatar University

## Abstract

In Qatar, one out of every ten patients admitted to the hospitals is in urgent need of a blood transfusion or blood products. The aims of this study are as follows: (1) to assess the level of awareness and knowledge about blood donation and (2) to identify the factors that contribute to the willingness to donate blood among young adults. A cross-sectional survey using a constructed questionnaire was conducted among students at Qatar University. A total of 590 responses were collected, out of which 423 were suitable for analysis. Only 72 out of 472 (15%) participants were blood donors. The chi-square test and *t*-test were then used to study the association of blood donation status with different factors. Significant values were considered to be *p* ≤ 0.5. Gender and age were found to be significantly associated with blood donation status, with a higher frequency of donation among males and adults above the age of 24 years old. On the other hand, the total knowledge score was found to not be significantly associated with blood donation status with a mean score of 60.5% for both groups (blood donors, non-blood donors). The most common motivators that encouraged blood donors were donating to help people, followed by having a blood mobile unit come to your place, whereas the most common barriers reported by non-blood donors were failing to meet the requirements, followed by “never having been asked to give blood”. This is the first study in Qatar to assess blood donation status. It provides insights that would help in developing effective strategies for the recruitment and retention of young adult blood donors in Qatar and countries with similar cultures. Raising awareness about blood donation, along with providing more mobile blood donation units at public places, will aid in increasing the frequency of blood donation among young adults.

## 1. Introduction

According to Hamad Medical Corporation (HMC), the main healthcare provider in Qatar, one out of every ten patients admitted to the hospital is in urgent need of a blood transfusion or blood products [1]. Blood is an essential component needed in healthcare facilities to save lives in a variety of circumstances, including traumas, surgeries, blood disorders, transplantations, pregnancy complications, and many other diseases [2]. Blood has no alternative source other than humans; it is mainly provided by involuntary blood donors (replacement for relatives or friends whose blood is unsuitable for the patient) and voluntary blood donors. The blood donation process is defined as a medical procedure that involves transferring blood from a healthy, voluntary person to someone who needs blood. There are many types of blood donations: whole blood, platelet, plasma, and red blood cells [3]. Each unit of blood donated (450 mL) can benefit at least three lives of people as separate components. Therefore, efforts continue worldwide to maintain a satisfactory number of blood donors to guarantee a sufficient, safe and timely blood supply that meets the clinical demand [4,5].

Due to the rise of chronic diseases, surgical procedures, traumas, cancers and road accidents, urgent fresh blood is needed, especially during the first 24 h of treatment [6]. Blood can only be stored for a limited time. Due to this, there is an urgent need for a regular blood supply to be available, once needed, at the right time, place and of the right blood type. Unfortunately, out of 38% of eligible blood donors, only 10% donate blood [7]. Therefore, many countries have been facing a shortage of blood donations and, consequently, do not meet the current clinical demand. This is most prominent in developed countries that have a lot of advances in the medical field and surgical procedures. In Qatar, the Qatar Blood Donor Center is the only public institution providing blood to all public and private hospitals and it is managed by HMC. In 1987, HMC stopped importing blood and blood components from outside the country and now completely relies on local blood donations [8].

The imbalance between blood supply and demand requires continuous efforts to develop new strategies and frameworks that will aid in recruiting more blood donors. Therefore, understanding the awareness and knowledge levels, motivators and barriers for blood donation is very important to establish successful strategies, campaigns and promotions [9,10]. Many studies and blood recruitment strategies have focused specifically on young adult groups because older people are more likely to need blood transfusions in the next few years. Thus, targeting young adults is extremely important to ensure an adequate blood supply for the next few years [11]. To achieve this, knowledge is an important factor to assess for blood donation as it is believed that people are more motivated to donate blood when they are well informed about the facts, myths and fears related to blood donation [12]. Other motivators for donating blood include altruism (helping patients), reluctant altruism (being pressured by society), subjective norms (being influenced by friends), reciprocity (availability for self, family or friends), incentives and curiosity [13]. On the other hand, the barriers and obstacles to donate blood include medical reasons, fear (needles, feeling dizzy, etc.), lifestyle barriers, lack of marketing communication, lack of knowledge about donating and negative experiences relating to blood [14].

Factors that contribute to the willingness to donate blood have been widely investigated; the outcome may vary from country to country. This is due to differences in traditions, culture, religion and level of education. Sociodemographic variables have also been reported to impact blood donation. In a study from Saudi Arabia, gender was found to influence the willingness to donate [15]. Moreover, age and level of education have also been found to have an effect on people’s attitude toward blood donation [16]. Therefore, in our survey, we examined different factors that contribute to blood donation. We also tried to understand the motivators and barriers that influence blood donation. To our knowledge, this is the first study in Qatar that aims to assess knowledge level and identify the motives and barriers that affect the willingness to donate blood among young adults at Qatar University. Our sample was not designed to be representative of the entire Qatari population, rather it is a convenient sample to help understand the determinants of blood donation in the education sector in Qatar.

## 2. Materials and Method

### 2.1. Study Design and Sampling Technique

A descriptive cross-sectional study was conducted among students at Qatar University. Data were collected in a two-week period during March 2020. Students were recruited randomly using a convenience sampling method by sending an online link to 8000 registered students to access the questionnaire through email announcements. Cochran’s sample size formula was used to ensure the sample size was representative. It was estimated that there are 357 participants with a 95% confidence interval and 5% margin of error.

### 2.2. Ethical Approval

The Institutional Review Board (IRB) of Qatar University has reviewed and approved this study, prior to initiation (approval number: QU-IRB 1268-EA/20). Participation in this study is voluntary, and an electronic online informed consent form was obtained from each participant.

### 2.3. Questionnaire

The questionnaire used in this study is an online self-administered questionnaire using Survey Monkey online software [17]. The content of this survey was adapted from previously published validated questionnaires [15,18,19,20], then it was translated into the Arabic language (Supplementary Materials). A pilot study using face-to-face interviews with the proposed questions in the survey was conducted before the actual data collection on 10 participants to evaluate the feasibility and readability of the items for targeted respondents. Pilot study participants were recruited from Qatar University students through social networks [21]. Using the comments given by the participants in the pilot study, the questions were reformulated for the ease of understanding. Participants that were included in the pilot study were excluded from the main study. The constructed questionnaire consists of four sections, with a total of 40 questions, of which four are open ended questions. The four sections are described below.

#### 2.3.1. Part A: Sociodemographic Information and Items Associated with Blood Donation Status (8 Items)

Items included age, gender, nationality, current academic year, college and three items associated with blood donation status. Respondents were classified into two groups in this section according to their donation status. The first group consisted of blood donors, who have donated blood one time or more, and the second group consisted of non-donors, who have never donated blood.

#### 2.3.2. Part B: Motivators to Donate Blood (9 Items)

This section was intended for blood donors only and used to assess the motivators to donate blood. Intentions for donating blood include altruism, reluctant altruism, subjective norms, reciprocity, incentives, curiosity and others as an open-ended question. These motivators were addressed using a set of questions and classified into two categories, which are motivators and facilitators, and responses were given using a 5-point Likert scale ranging from “Strongly agree” to “Strongly disagree”.

#### 2.3.3. Part C: Barriers to Donate Blood (11 Items)

This section was intended for non-donors only and used to assess barriers for blood donation. Reasons for not donating blood include low self-efficacy, fear, inconvenience, lack of knowledge, negative attitudes and others as an open-ended question. The barriers were addressed using a set of questions, and responses were given using a 5-point Likert scale ranging from “Strongly agree” to “Strongly disagree”.

#### 2.3.4. Part D: Knowledge Assessment on Blood Donation (12 Items)

The knowledge assessment questions were constructed based on a validated survey [20]. The content validity ratio (CVR) was calculated for 24 items; of those, only the 12 items with the highest CVR were included. It consists of 12 items that require true or false responses.

### 2.4. Data Analysis

Results of the questionnaire were extracted using Excel and then imported and analyzed using SPSS version 26 (IBM^®^ Statistics, Chicago, IL, USA). Significant associations between sociodemographic variables and blood donation status were examined using a chi-squared test, since these are two categorical variables. A chi-squared test was also used to assess the association of each individual’s answer with their donation status, in a knowledge assessment quiz. Regarding the knowledge assessment, a reliability test for the 12 items was performed using Cronbach’s Alpha. An independent *t*-test was used to compare the mean knowledge score of blood donors and non-blood donors to determine if there is any association between knowledge level and the action of donating blood. Frequency calculations were used to analyze barriers and motivators; each item of the barriers and motivators were analyzed in terms of association with age and gender using a chi-squared test. A *p*-value ≤ 0.05 was considered statistically significant.

## 3. Results

Out of the total 590 questionnaire responses collected from students at Qatar University, 118 were excluded due to incomplete responses. This resulted in a total of 472 complete responses for analysis (80% completion rate). The majority of the respondents were female (79.5%), and only 20.9% were male. Among the respondents, 72 (15.3%) had donated blood at least once in their lifetime and 400 were non-blood donors. To measure the frequency of donation during the last year only, 49 (71.0%) had donated blood only once, 12 (17.4%) had donated twice, 7 (10.1%) had donated three to four times and 1 (1.45%) had donated more than five times.

### 3.1. Sociodemographic Characteristics and Association with Blood Donation Status

We found an association between gender (*p* < 0.001) and age (*p* = 0.002) with the donation status (Table 1). More males than females are likely to donate blood (35.1% vs. 10.1%) and this proportion difference is statistically significant. Additionally, the majority of age groups older than 24 years in both genders were more likely to donate blood (26.8%), compared to the other age groups.

### 3.2. Knowledge Level toward Blood Donation

The knowledge assessment consisted of 12 items, which was tested for reliability using Cronbach’s alpha. Cronbach’s Alpha was α = 0.606, which is considered to be acceptable. The blood donors had a higher mean knowledge score (M = 63.9%, SD = 19.8) than the non-blood donors (M = 59.8%, SD = 20.0) (Table 2). However, an independent *t*-test indicates that this difference was not significant *t*(470) = 1.560, *p* > 0.05 (Table 2). The most common ways of hearing about blood donation, selected by most participants, are social media (71.2%) and relatives/friends (58.6%).

### 3.3. Motivators/Facilitators of dDnating Blood (Donors)

**Figure 1:**
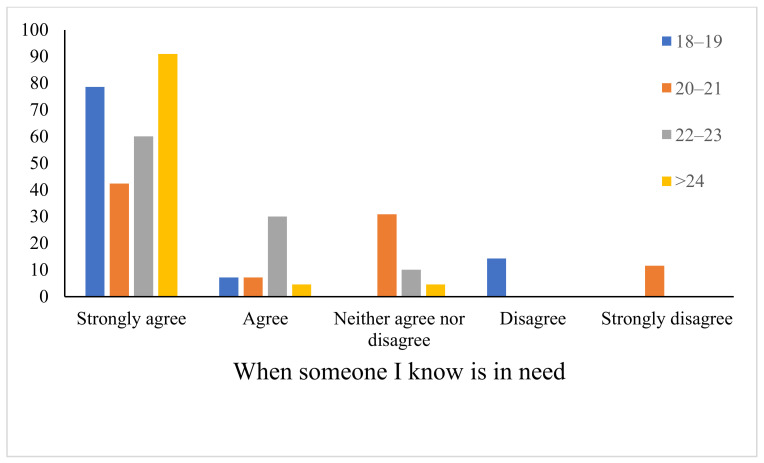
Association between age and blood donation motivators (When someone I know is in need) (*n* = 72).

The most common motivators reported by blood donors are donating to help patients (87.5% strongly agree, 9.7% agree), having a mobile blood donation unit come to your place (63.9% strongly agree, 19.4% agree) and when someone I know is in need (66.7% strongly agree, 12.5% agree) (Table 3). The results showed significant associations between age and “when someone I know is in need” (Figure 1 and Table 4). Blood donors older than 24 years agreed more than others that they have donated blood previously when someone they know is in need (90.9% strongly agree) (Figure 1). No significant associations were found between any of the motivators and gender (Table 4).

### 3.4. Barriers of Donating Blood (Non-Blood Donors)

**Figure 2:**
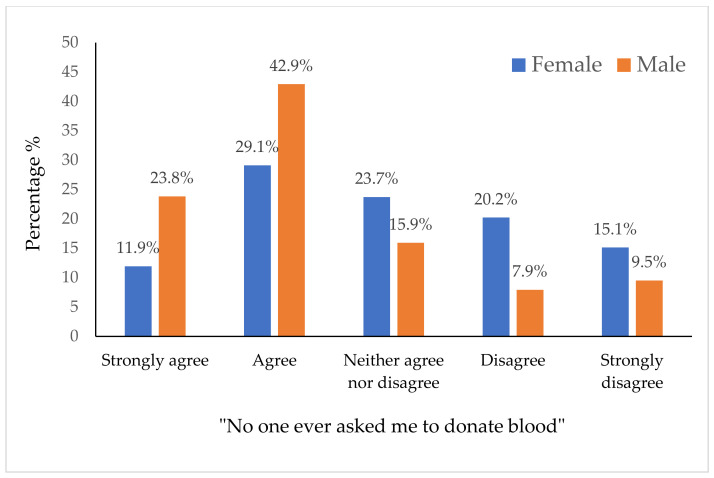
Association between gender and blood donation barriers (“No one ever asked me to donate blood”) (*n* = 400).

The most common barriers reported by non-blood donors are failing to meet the requirements (26.3% strongly agree, 18.8% agree), and “no one ever asked me to give blood” (13.8% strongly agree, 31.3% agree) (Table 5). In addition, significant associations were found between gender and “I do not think there is a need to donate blood”, “no one ever asked me to donate blood” and “failing to meet the requirement” (Table 6). No significant associations were found between any of the barriers and age. More males than females agreed to no one ever asked me to donate blood (males: 23.8% strongly agree, 42.9% agree versus females: 11.9% strongly agree, 29.1% agree) (Figure 2).

### 3.5. Effective Ways of Promoting Blood Donation

The most effective ways of promoting blood donation selected by all the participants are raising the awareness of blood donation (81.3%), followed by providing a mobile blood donation system (66.2%), and developing a mobile technology for blood donation (62.1%). Most of the participants agreed that developing a mobile application would help in promoting blood donation (49.4% strongly agree, 33.7% agree), and selected that the most helpful feature of a mobile application is viewing the locations of the closest donation center or mobile donation unit (84.5%).

## 4. Discussion

In this study, we aimed to identify factors that contribute to the willingness to donate blood among students at Qatar University. In our survey, 472 students participated, of which only 15% were blood donors. Our results revealed gender to have a significant association with the participant’s blood donation status. In line with previous studies, many countries (United States, Saudi Arabia and India) have also reported a higher percentage of male donors than females [15,22,23]. However, contradictory findings regarding the association between gender and blood donation status have been reported in China [11]. A systematic review of 80 publications regarding gender variation in giving blood found that although women are more altruistic than men, they donate blood less frequently due to low levels of hemoglobin, menstrual cycle, weight requirements and adverse reactions such as dizziness [24]. The contradicting findings reported in China might be because they allow woman during their menstrual cycle to donate blood as long as they have a normal hemoglobin level. The human sex ratio, which is the number of males for each female in a country’s population, in Qatar is 1.02 upon birth; however, it is 2.64 for the 15–24 age brackets [25]. This is mainly influenced by a huge influx of male expatriates. In the light of all the above, it is important to tailor recruitment campaigns to close the gender gap in blood donation.

We found that age is significantly associated with blood donation status, where a higher percentage of students older than 24 years were blood donors, compared to younger students. This has also been reported in China among college students. The reasons for this association have been hypothesized to include the study load of students in the first few years of college and their knowledge level at the time [11]. Surprisingly, the total knowledge score of participants has not been found to be associated with blood donation status in our study. The knowledge means of blood donors and non-blood donors were very close, suggesting that knowledge level does not affect the decision of donating blood. These results were contradictory to what has been reported in previous studies, which highlighted the association between knowledge level and blood donation status [26,27,28]. Contradictory findings might be due to the small proportions of blood donors in our study, only 15% compared to other studies where blood donors account for more than 50% of the participants.

The most common motivators reported by blood donors is donating to help people, followed by having a blood mobile unit come to your place and lastly when someone I know is in need. This is in agreement with studies conducted in other countries including China, Turkey, Malaysia and Greece [11,18,29,30], where helping patients (referred to as altruism) has been highlighted as one the main reasons for donating blood. The presence of a mobile donation unit in public areas has been highlighted as one of the main motivators in one study in Saudi Arabia [12].

The most common barriers reported by non-blood donors are failing to meet the requirements followed by “no one ever asked me to give blood”. Both barriers were significantly associated with gender; however, failing to meet the requirements has been reported more frequently by females, while “no one asked me to give blood” was more frequently reported by males, which has also been reported by other studies [31]. This question was actually adapted from a study in the USA [19]; however, in their study only 16% of non-donors agreed to this statement compared to 45% in our study. The findings in our study may be due to poor awareness about the blood donation requirements in Qatar, which has already been proven in the knowledge assessment section. It is important to highlight that no published studies have been found about the awareness of blood donation in Qatar, and most of the non-donors reported that they have never been asked to donate blood.

We sought to understand the preferred way of promoting blood donation among young adults to provide guidelines and insights that would help in addressing this issue and developing an effective recruitment system for young adult donors in Qatar. Raising the awareness and benefit of blood donation is the most effective way that was selected by our participants, followed by providing mobile collection venues, which have been shown to be more successful in donor recruitment [32]. Another effective promotion selected by participants is a mobile phone application, which has not yet been developed in Qatar. Mobile applications for blood donation with the important features for the recruitment and retention of blood donors have proven to be successful in many countries [33,34,35]. Some other suggestions showed that some students are lacking the necessary information about the blood donation system in Qatar. For example, one student suggested in an open-ended question to lengthen the hours of the blood donation center at HMC instead of mornings only; although, the blood donation center opens until 9:00 p.m. every day. This emphasizes the need to increase the level of awareness of blood donation system in Qatar among university students.

Blood donation is a lifesaving therapy. Our study showed that the presence of mobile collection venues at the university will help to increase the number of donors. Moreover, developing a mobile phone application would facilitate effective communication with blood donors to encourage attendance [36]. A recent large study, called the INTERVAL study, carried out at Cambridge and Oxford Universities, which involved 45,000 blood donors, found that some blood donors could safely give blood more frequently than is allowed at present [37]. This new finding will allow policy makers the option to allow more frequent collection from donors. An increase in awareness and communication will increase the possibility of more potential donors. Consequently, the blood supply will increase, providing great clinical support for the healthcare system in Qatar. This would make managing the reserve easy for the blood donation center and reduce the psychological pressure on the relatives of patients in need [38].

### Limitations

The study has some limitations. First, we used a cross sectional study with a convince sampling method to include only young adults at Qatar University. An extension to this study could focus on the alternate age brackets. Although the design and sampling method serves our objective, one might be cautioned that the findings cannot be generalizable to all young adults in Qatar. Another limitation is the low response rate of male participants compared to females. This can be explained by the low number of male students at Qatar University, where 80% of the students are female. Another limitation is that the motivators were only assessed for blood donors and, thus, cannot be representative of those of non-donors. We measured the frequency of donation only during the last year. It would be of interest to measure the frequency of donation over a longer period of time to identify any decline in blood donation. Further studies may include participants from different universities, and motivators can also be assessed for non-donors.

## 5. Conclusions

In summary, assessing the knowledge level of both blood donors and non-blood donors and understanding the motives and barriers for blood donation is critical to develop effective strategies to recruit more donors to meet the clinical need. Interestingly, the current study revealed that knowledge level was not associated with donation status. Altruism and the presence of mobile donation units were the main motivators for donating blood among blood donors. These findings suggest that raising the awareness about blood donation among young adults in Qatar may increase blood donation frequency. Future work could focus on younger age groups to understand more about their motivation. More efforts should be made in using social media and influencers to raise awareness about blood donation, since it has been indicated in this survey that participants have heard about blood donation before through those methods. Therefore, it merits further consideration and research to recruit future donors.

## Figures and Tables

**Table 1:** Univariate association between sociodemographic characteristics donor status of respondents (*N* = 472).

Variable	Total (*n* = 472)	Non-Blood Donor (*n* = 400)	Blood Donor (*n* = 72)	*X* ^2^	*p*	df
Gender						
Male	97 (20.6%)	63 (64.9%)	34 (35.1%)	37.016	<0.001 *	1
Female	375 (79.4%)	337 (89.9%)	38 (10.1%)			
Age						
18–19	168 (35.6)	154 (91.7%)	14 (8.3%)	14.946	0.002 *	3
20–21	164 (34.7%)	138 (84.1%)	26 (15.9%)			
22–23	58 (12.3%)	38 (82.8%%)	10 (17.2%)			
>24	82 (17.4%)	60 (73.2%)	22 (26.8%)			
Nationality						
Qatari	225 (47.7%)	187 (83.1%)	38 (16.9%)	0.889	0.346	1
Non-Qatari	247 (52.3%)	213 (86.2%)	34 (13.8%)			
Academic Year						
Freshman	120 (25.4%)	103 (85.8%)	17 (14.2%)	5.683	0.224	4
Sophomore	108 (22.9%)	96 (88.9%)	12 (11,1%)			
Junior	89 (18.9%)	73 (82.0%)	16 (18.0%)			
Senior	112 (23.7%)	96 (85.7%)	16 (14.3%)			
Graduate student	43 (9.1%)	32 (74.4%)	11 (25.6%)			
Major						
Non-Health related	355 (75.2%)	299 (84.2%)	56 (15.8%)	0.300	0.584	1
Health related	117 (24.8%)	101 (86.3%)	16 (13.7%)			

* Significant *p*-value (≤0.05), *X*^2^: Chi-square, df: degree of freedom.

**Table 2:** Knowledge level toward blood donation among blood donors and non-blood donors.

Questions	Total (*n* = 472)	Donors (*n* = 72)	Non-Donors (*n* = 400)	*p*
1. Do you know your blood type?				
Yes-	433 (91.7%)	71 (98.6%)	362 (90.5%)	0.021 *
No	39 (8.3%)	1 (1.4%)	38 (9.5%)	
2. Can a donor be infected by donating blood?				
Yes	192 (40.7%)	37 (51.4%)	155 (38.8%)	0.130 *
No-	147 (31.1%)	19 (26.4%)	128 (32.0%)	
I do not know	133 (28.2%)	16 (22.2%)	117 (29.3%)	
3. Will your blood be tested before transfusing it to other people?				
Yes-	389 (82.4%)	62 (86.1%)	327 (81.8%)	0.557 *
No	11 (2.3%)	2 (2.8%)	9 (2.3)	
I do not know	72 (15.3)	8 (11.1%)	64 (16.0)	
4. When someone donates blood, does the blood volume return to normal level within 24–48 h?				
Yes-	216 (45.8%)	31 (43.1%)	185 (46.3%)	
No	47 (10.0%)	11 (15.3)	36 (9.0%)	0.262 *
I do not know	209 (44.3%)	30 (41.7%)	179 (44.8%)	
5. How long does the donation process take once the person enters the donation room?				
20 min-	225 (47.7%)	52 (72.2%)	173 (43.3%)	
30 min to 1 h	76 (16.1%)	13 (18.1%)	63 (15.8%)	<0.001 *
More than 1 h	4 (0.8%)	1 (1.4%)	3 (0.8%)	
I do not know	167 (35.4%)	6 (8.3)	161 (40.3%)	
6. Should the blood donor be fasting?				
Yes	82 (17.4%)	10 (13.9%)	72 (18.0%)	
No-	233 (49.4%)	55 (76.4%)	178 (44.5%)	<0.001 *
I do not know	157 (33.3%)	7 (9.7%)	150 (37.5%)	
7. Can a person with diabetes or high blood pressure donate blood?				
Yes	47 (10.0%)	12 (16.7%)	35 (8.8%)	
No-	309 (65.5%)	39 (54.2%)	270 (67.5%)	0.044 *
I do not know	116 (24.6%)	21 (29.2%)	95 (23.8%)	
8. Can a person with fever donate blood?				
Yes	30 (6.4%)	6 (8.3%)	24 (6.0%)	
No-	306 (64.8%)	54 (75.0%)	252 (63.0%)	0.044 *
I do not know	136 (28.8%)	12 (16.7%)	124 (31.0%)	
9. Can a pregnant woman donate blood?				
Yes	12 (2.5%)	3 (4.2%)	9 (2.3%)	
No-	350 (74.2%)	46 (63.9%)	304 (76.0%)	0.090 *
I do not know	110 (23.3%)	23 (31.9%)	87 (21.8%)	
10. Can a menstruating woman donate blood?				
Yes	42 (8.9%)	7 (9.7%)	35 (8.8%)	
No-	281 (59.5%)	49 (68.1%)	232 (58.0%)	0.178 *
I do not know	149 (31.6%)	16 (22.2%)	133 (33.3%)	
11. Can a breastfeeding woman donate blood?				
Yes	48 (10.2%)	16 (22.2%)	32 (8.0%)	
No-	264 (55.9%)	33 (45.8%)	231 (57.8%)	<0.001 *
I do not know	160 (33.9%)	23 (31.9%)	137 (34.3%)	
12. Can blood be stored for more than 24 h if not used immediately?				
Yes-	274 (58.1%)	41 (56.9%)	233 (58.3%)	
No	26 (5.5%)	8 (11.1%)	18 (4.5%)	0.070
I do not know	172 (36.4%)	23 (31.9%)	149 (37.3%)	
Knowledge mean score	60.5%	63.9%	59.8%	t(470) = 1.560 ** p = 0.119 95% CI: 1.04 to 9.02
Standard deviation	20.0	19.8	20.0	
Highest score	100%	100%	100%	
Lowest score	0%	8.33%	0%	

- correct response. * chi-squared test. ** independent *t*-test.

**Table 3:** Motivators/facilitators toward blood donation (*N* = 72).

Items		Strongly Agree (%)	Agree (%)	Neither Agree nor Disagree (%)	Disagree (%)	Strongly Disagree (%)
Motivators	(a) Donating to help patients	87.5%	9.7%	2.8%	0%	0%
	(b) When someone I know is in need	66.7%	12.5%	13.9%	2.8%	4.2%
	(c) Friends or family who are donors had an influence on me	29.2%	22.2%	23.6%	11.1%	13.9%
Facilitators	(d) Incentives for donation (free gifts, food, vacation)	11.1%	6.9%	16.7%	20.8%	44.1%
	(e) Free health check	15.3%	22.2%	19.4%	15.3%	27.8%
	(f) The place of blood donation center is convenient	31.9%	30.6%	25.0%	6.9%	5.6%
	(g) Convenient working hours of blood donation center	23.6%	27.8%	37.5%	6.9%	4.2%
	(h) Having a blood mobile unit come to your place of work or other place	63.9%	19.4%	11.1%	1.4%	4.2%
	(i) Others (open-ended question)	1.7%				

**Table 4:** Association between Gender/Age and Blood Donation Motivators.

Items	*p*-Value (Gender)	*p*-Value (Age)
(a) Donating to help patients	0.113	0.097
(b) When someone I know is in need	0.307	<0.002 *
(c) Friends or family who are donors had an influence on me	0.699	0.783
(d) Incentives for donation (free gifts, food, vacation)	0.153	0.202
(e) Free health check	0.237	0.590
(f) The place of blood donation center is convenient	0.553	0.517
(g) Convenient working hours of blood donation center	0.848	0.951
(h) Having a blood mobile unit come to your place of work or other place	0.454	0.545

* Significant *p*-value (≤0.05).

**Table 5:** Barriers toward blood donation (*N* = 400).

Items	Strongly Agree (%)	Agree (%)	Neither Agree nor Disagree (%)	Disagree (%)	Strongly Disagree (%)
(a) I do not think there is a need to donate blood	1.3%	2.0%	7.0%	26.3%	63.5%
(b) I might get HIV or AIDS from giving blood	1.5%	8.8%	20.0%	26.5%	43.5%
(c) No one ever asked me to give blood	13.8%	31.3%	22.5%	18.3%	14.2%
(d) Failing to meet the requirements (body weight, blood pressure, hemoglobin, etc.)	26.3%	18.8%	19.3%	18.3%	17.5%
(e) Fear (needles, feeling dizzy, etc.)	12.8%	19.8%	9.8%	26.0%	31.8%
(f) I do not have time to donate blood	7.5%	17.8%	24.3%	24.5%	26.0%
(g) I do not know where to donate blood	10.5%	21.8%	12.3%	26.5%	29.0%
(h) Inconvenient hours for blood donations sites	5.3%	11.8%	36.3%	23.3%	23.5%
(i) Inconvenient locations for blood donations sites	6.5%	14.2%	31.5%	24.5%	23.3%
(j) Limitation of activities after donation	2.8%	9.5%	35.8%	26.0%	26.0%
(k) Others (open-ended question)	11.7%				

**Table 6:** Association between gender/age and blood donation barriers.

Items	*p*-Value (Gender)	*p*-Value (Age)
(a) I do not think there is a need to donate blood	<0.008 *	0.063
(b) I might get HIV or AIDS from giving blood	0.676	0.554
(c) No one ever asked me to give blood	<0.003 *	0.924
(d) Failing to meet the requirements (body weight, blood pressure, hemoglobin, etc.)	<0.005 *	0.504
(e) Fear (needles, feeling dizzy, etc.)	0.381	0.805
(f) I do not have time to donate blood	0.782	0.579
(g) I do not know where to donate blood	0.125	0.493
(h) Inconvenient hours for blood donations sites	0.273	0.495
(i) Inconvenient locations for blood donations sites	0.446	0.929
(j) Limitation of activities after donation	0.192	0.556

* Significant *p*-value (≤0.05).

## Data Availability

Not applicable.

## Supplementary Materials

The following are available online at https://www.mdpi.com/article/10.3390/healthcare9080926/s1, Supplementary 1: Questionnaire in English and Arabic.
