# Supplementary material for: Knowledge Level, Motivators and Barriers of Blood Donation among Students at Qatar University
Source: Healthcare (Basel). 2021 Jul 22;9(8):926. doi: 10.3390/healthcare9080926 (PMC8391523; doi:10.3390/healthcare9080926)
Supplement: Supplementary file 1 [file healthcare-09-00926-s001.zip › healthcare-1182111-supplementary.pdf]

healthcare MDPI

Questionaire in English/Arabic

- **Part A** (sociodemographic and blood donation status):

الجزء أ: المعلومات الاجتماعية والديموغرافية وحالة التبرع بالدم (8 بنود)

1. What is your age?

ما هو عمرك

- o 18–19
- o 20–21
- o 22–23
- o 24 or above أو أكثر

2. Gender

الجنس

- o Male ذكر
- o Female أنثى

3. Nationality

الجنسية

- o Select nationality اختر جنسيتك

4. Current academic year

العام الدراسي الحالي

- o Freshman (Credit Hours < 29)

طالب سنة أولى (الساعات المكتسبة<29)

- o Sophomore (Credit Hours 30–59)

طالب سنة ثانية (الساعات المكتسبة 30-59)

- o Junior (Credit Hours 60–89)

طالب سنة ثالثة (الساعات المكتسبة 60-89)

- o Senior (Credit Hours > 90)

طالب سنة رابعة (الساعات المكتسبة>90)

- o Graduate student

طالب دراسات عليا

5. College

الكلية

- o College of Arts and Science كلية الآداب والعلوم
- o College of Business and Economics كلية الإدارة والاقتصاد
- o College of Education كلية التربية
- o College of Engineering كلية الهندسة
- o College of Law كلية القانون

- College of Sharia and Islamic Studies كلية الشريعة والدراسات الإسلامية
- College of Health Sciences كلية العلوم الصحية
- College of Medicine كلية الطب
- College of Pharmacy كلية الصيدلة
- College of Dental Medicine كلية طب الأسنان

6. Where did you hear about blood donation? Participants could provide more than one answer in this question.

أين سمعت عن التبرع بالدم؟ من ممكن اختيار أكثر من إجابة واحدة في هذا السؤال.

- ☐ Friends/relatives الأصدقاء / الأقارب
- ☐ Television /Radio /Newspaper التلفزيون/ الراديو/ جريدة الأخبار
- ☐ Social media وسائل التواصل الاجتماعي
- ☐ Awareness campaign at university حملة توعية بالجامعة
- ☐ Awareness campaign or advertisement at shopping mall حملة توعية أو إعلان في مجمع تجاري
- ☐ Educational lecture محاضرة تثقيفية
- ☐ I have never heard of blood donation لم أسمع بالتبرع بالدم من قبل
- ☐ Others, please specify الأخرى، يرجى تحديد ذلك

7. Have you ever donated blood before?

هل تبرعت بالدم من قبل؟

- Yes نعم
- No لا

8. If yes, how many times in the last one year?

إذا كانت الإجابة نعم، كم مرة قد تبرعت بالدم خلال العام الماضي؟

- Once مرة واحدة
- Twice مرتين
- Three to four times ثلاث إلى أربع مرات
- More than five times أكثر من خمس مرات

- **Part B** (Motivators- only for blood donors):

الجزء ب :المحفزات للتبرع بالدم (8 بنود) – الإجابة من قبل بالدم فقط

9. What motivated you to donate blood?

ما الذي دفعك أو حفزك للتبرع بالدم؟

Item	Strongly agree أوافق بشدة	Agree أوافق	Neither agree nor disagree لا أوافق ولا أرفض	Disagree أرفض	Strongly disagree أرفض بشدة
a) Donating to help patients التبرع لمساعدة المرضى					
b) When someone I know is in need عندما يكون شخص أعرفه في حاجة					
c) Friends or family who are donors had influence on me					

عائلتي أو أصدقائي المتبرعين بالدم كان لهم تأثير علي					
d) Incentives for donation (free gifts, food, vacation)<br>مكافئات التبرع بالدم (هدايا مجانية، طعام، عطلة من العمل)					
e) Free health check<br>فحص طبي مجاني					
f) The place of blood donation center is convenient<br>مكان مركز التبرع بالدم مناسب					
g) Convenient working hours of blood donation center<br>ساعات العمل في مركز التبرع بالدم مناسبة					
h) Having a blood mobile unit come to your place of work or other place<br>وجود وحدة متنقلة للدم تأتي إلى مكان عملك أو أي مكان آخر					

i) Other motivators, please specify? دوافع أخرى، يرجى التحديد؟

- Part C (Barriers- only for non- donors):

الجزء ت: الحواجز التي تحول دون التبرع بالدم (9 بنود) – الإجابة من قبل غير المتبرعين بالدم فقط

10. What prevented you from donating blood?

ما الذي يمنعك من التبرع بالدم؟

Item	Strongly agree<br>أوافق بشدة	Agree<br>أوافق	Neither agree nor disagree<br>لا أوافق ولا أرفض	Disagree<br>أرفض	Strongly disagree<br>أرفض بشدة
a) I do not think there is a need to donate blood<br>لا أعتقد هناك حاجة للتبرع بالدم					
b) I might get HIV or AIDS from giving blood<br>قد أصاب بالإيدز أو فايروس نقص المناعة نتيجة التبرع بالدم					
c) No one ever asked me to give blood<br>لم يطلب أحد مني التبرع بالدم					
d) Failing to meet the requirements (body weight, blood pressure, hemoglobin, etc.)<br>الفشل في تلبية متطلبات التبرع بالدم (الوزن، ضغط الدم، مستوى الهيموغلوبين)					
e) Fear (needles, feeling dizzy, etc.)<br>الخوف (الإبر ، الشعور بالدوار ، وما إلى ذلك)					
f) I do not have time to donate blood<br>ليس لدي وقت للتبرع بالدم					
g) I do not know where to donate blood<br>لا أعرف مكان التبرع بالدم					

h) Inconvenient hours for blood donations sites
ساعات العمل في مركز التبرع بالدم غير مناسبة
i) Inconvenient locations for blood donations sites
مكان مركز التبرع بالدم غير مناسب
j) Limitation of activities after donation
الأنشطة التي يمكنني أن أقوم بها بعد التبرع بالدم محدودة

k) Other barriers, please specify and write your feedback?
حواجز أخرى، يرجى التحديد؟

- **Part D** (knowledge assessment on blood donation):

الجزء ث: تقييم المعرفة حول التبرع بالدم (8 بنود)

1. Do you know your blood type?
هل تعرف فصيلة دمك؟
- o Yes نعم
- o No لا

2. Can a donor be infected by donating blood?
هل يمكن للعدوى أن تنتقل للمتبرع بالدم؟
- o Yes نعم
- o No لا *
- o I do not know لا أعلم

3. Will your blood be tested before transfusing it to other people?
هل سيتم فحص دمك من الأمراض قبل نقله إلى أشخاص آخرين؟
- o Yes نعم*
- o No لا
- o I do not know لا أعلم

4. When someone donates blood, does the blood volume return to normal level within 24–48 hours?
عندما يتبرع شخص بالدم، هل تعود كمية الدم في جسمه إلى ما كان عليه خلال 24-48 ساعة؟
- o Yes نعم*
- o No لا
- o I do not know لا أعلم

5. How long does the donation process take one the person enters the donation room?
كم تستغرق عملية التبرع بالدم بمجرد دخول شخص إلى غرفة التبرع للتبرع بالدم؟
- o 20 min 20 دقيقة*
- o 40 m to 1 hour 40 دقيقة إلى 1 ساعة
- o More than 1 hour أكثر من ساعة

- I don’t know لا أعلم

6. In order to donate blood, should the donor be fasting?
هل يجب على المتبرع بالدم أن يكون صائمًا؟
   - Yes نعم
   - No لا *
   - I do not know لا أعلم

7. Can the following donate blood?
هل من الممكن للأشخاص في الحالات التالية للتبرع بالدم؟
   a) a person who has diabetes or high blood pressure
الشخص المصاب بداء السكري أو ارتفاع ضغط الدم؟
   - Yes نعم
   - No لا *
   - I do not know لا أعلم

   b) A person who has fever?
الشخص الذي يعاني من الحمى؟
   - Yes نعم
   - No لا *
   - I do not know لا أعلم

   c) Pregnant woman
المرأة الحامل
   - Yes نعم
   - No لا *
   - I do not know لا أعلم

   d) Breastfeeding woman
المرأة المرضعة
   - Yes نعم
   - No لا *
   - I do not know لا أعلم

   e) Menstruating woman
المرأة في فترة الحيض
   - Yes نعم
   - No لا *
   - I do not know لا أعلم

8. Can blood be stored for more than 24 hours if not used immediately?
هل يمكن تخزين الدم إذا لم يتم استخدامه مباشرة؟
   - Yes نعم*
   - No لا
   - I do not know لا أعلم

- **Part E** (Promoting blood donation):

الجزء ج: الترويج للتبرع بالدم (4 بنود)

1. The most effective ways of promoting blood donation considered by all participants? Participants could provide more than one answer in this question.

بنظرك ما هي الطرق الأكثر فعالية للترويج بالتبرع بالدم؟ من الممكن اختيار أكثر من إجابة واحدة في هذا السؤال.

☐ Raising the awareness of blood donation
زيادة التوعية بالتبرع بالدم

☐ Increasing the values of incentives
زيادة المكافئات

☐ Increasing blood donation sites
زيادة مواقع التبرع بالدم

☐ Provide mobile blood donation system (i.e: truck)
توفير نظام متنقل للتبرع بالدم (أي: شاحنة)

☐ Lengthening the operation hours
إطالة ساعات العمل لمركز التبرع بالدم

☐ Recruit donors via social media
اتواصل مع المتبرعين بالدم عبر وسائل التواصل الاجتماعي

☐ Develop a mobile technology that send reminders to blood-donors when needed
تطوير تطبيق في الهاتف المحمول يقوم على ارسال رسائل تذكيرية إلى المتبرعين بالدم عند الحاجة

☐ Others, please specify
الأخرى، يرجى تحديد ذلك

2. Do you think a mobile application can help in promoting in blood donation?

هل تعتقد أن تطبيق هاتف محمول خاص بالتبرع بالدم يمكن أن يساعد في الترويج للتبرع بالدم؟

o Strongly agree أوافق بشدة
o Agree أوافق
o Neither agree nor disagree لا أوافق ولا أرفض
o Disagree أرفض
o Strongly disagree أرفض بشدة

3. If yes, what are the features that you believe are helpful in mobile application for blood donation? Participants could provide more than one answer in this question.

ما هي الميزات التي تعتقد أنها مفيدة في تطبيقات الهاتف المحمول للتبرع بالدم؟

☐ View locations of the closest donation center or mobile unit
عرض أقرب موقع مركز للتبرع بالدم أو محطة متنقلة

☐ Schedule appointments
حجز موعد

☐ Request pickup to donate blood
طلب خدمة توصيل إلى مركز التبرع بالدم

☐ Fill donor questionnaire before going to blood donation center to speed up the process

ملئ استبيان التبرع بالدم قبل الذهاب إلى مركز التبرع بالدم لتسريع العملية

☐ Send notifications when there is a need for specific blood group that matches the donor blood group.

ارسال إشعارات عندما تكون هناك حاجة إلى فصيلة دم معينة تتطابق مع فصيلة دمي

☐ Information on blood donation process

معلومات عن عملية التبرع بالدم

☐ Others, please specify الأخرى يرجى التحديد
